# Supplementary material for: DHODH Inhibition Suppresses MYC and Inhibits the Growth of Medulloblastoma in a Novel In Vivo Zebrafish Model
Source: Cancers (Basel). 2024 Dec 13;16(24):4162. doi: 10.3390/cancers16244162 (PMC11674817; doi:10.3390/cancers16244162)
Supplement: Supplementary file 1 [file cancers-16-04162-s001.zip › cancers-3289340-supplementary.pdf]

Supplementary Materials: DHODH Inhibition Suppresses *MYC* and Inhibits the Growth of Medulloblastoma in a Novel In Vivo Zebrafish Model

Ioanna Tsea, Thale Kristin Olsen, Panagiotis Alkinoos Polychronopoulos, Conny Tümmler, David B. Sykes, Ninib Baryawno and Cecilia Dyberg

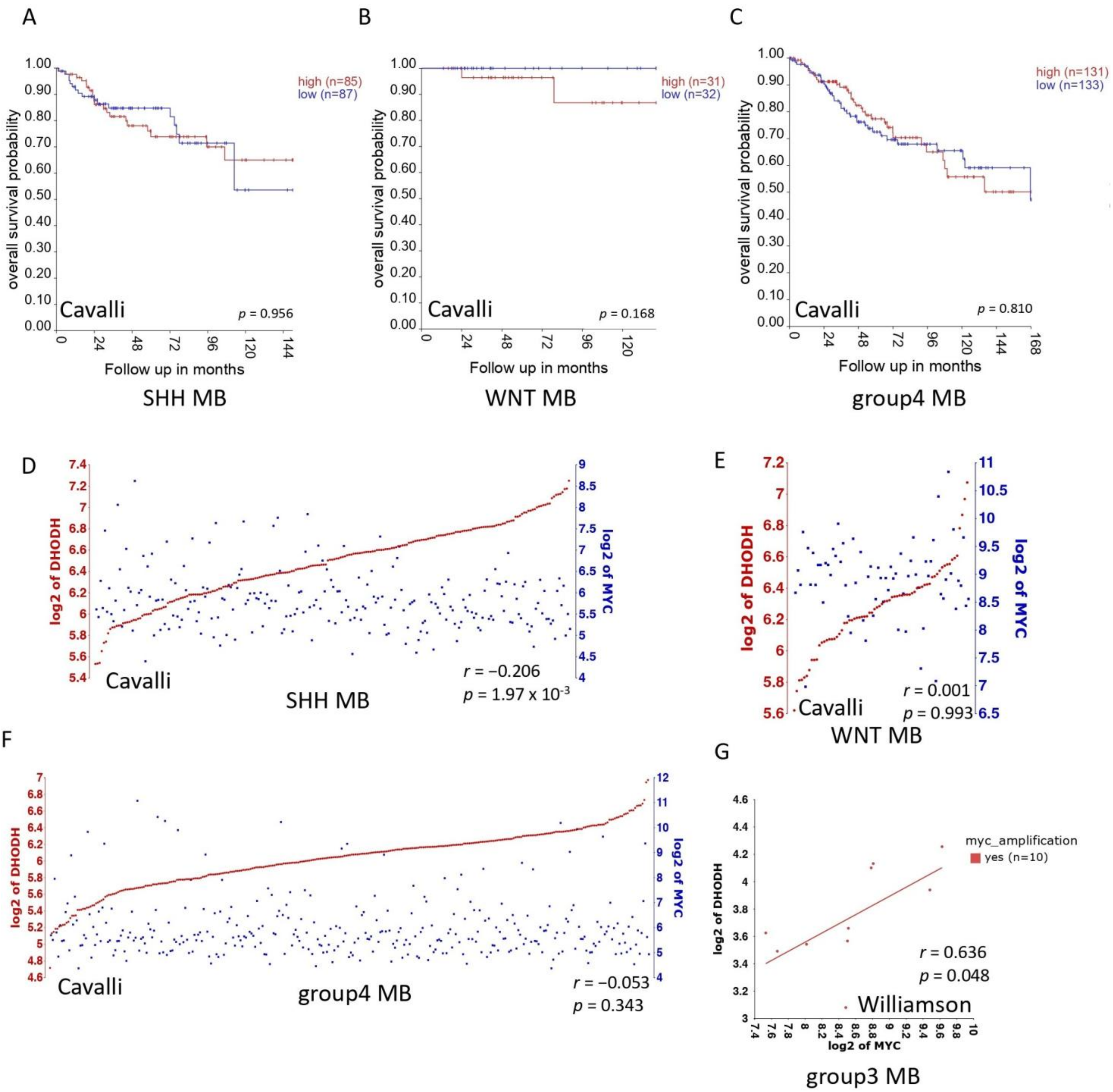

Figure S1. *DHODH* is expressed in medulloblastoma tumours. (**A**–**C**) Overall survival of SHH, WNT and group 4 medulloblastoma patients separated by median *DHODH* expression using the Cavalli dataset $n = 763$ primary medulloblastomas including SHH $n = 172$, WNT $n = 63$ and group 4 $n = 264$. Red, *DHODH* above median (high); Blue, *DHODH* below median (low). Groups are compared using log-rank test. (**D**–**F**) Correlation of *DHODH* with *MYC* in SHH (D), WNT (E) and group 4 (F) medulloblastoma patients using the Cavalli dataset $n = 763$ primary medulloblastomas including SHH $n = 223$, Wnt $n = 70$ and group 4 $n = 326$. (**G**) Correlation of *DHODH* with *MYC* in group 3 medulloblastoma patients showing MYC amplification using the Williamson dataset $n = 10$ Group 3 primary medulloblastomas with *MYC* amplification.

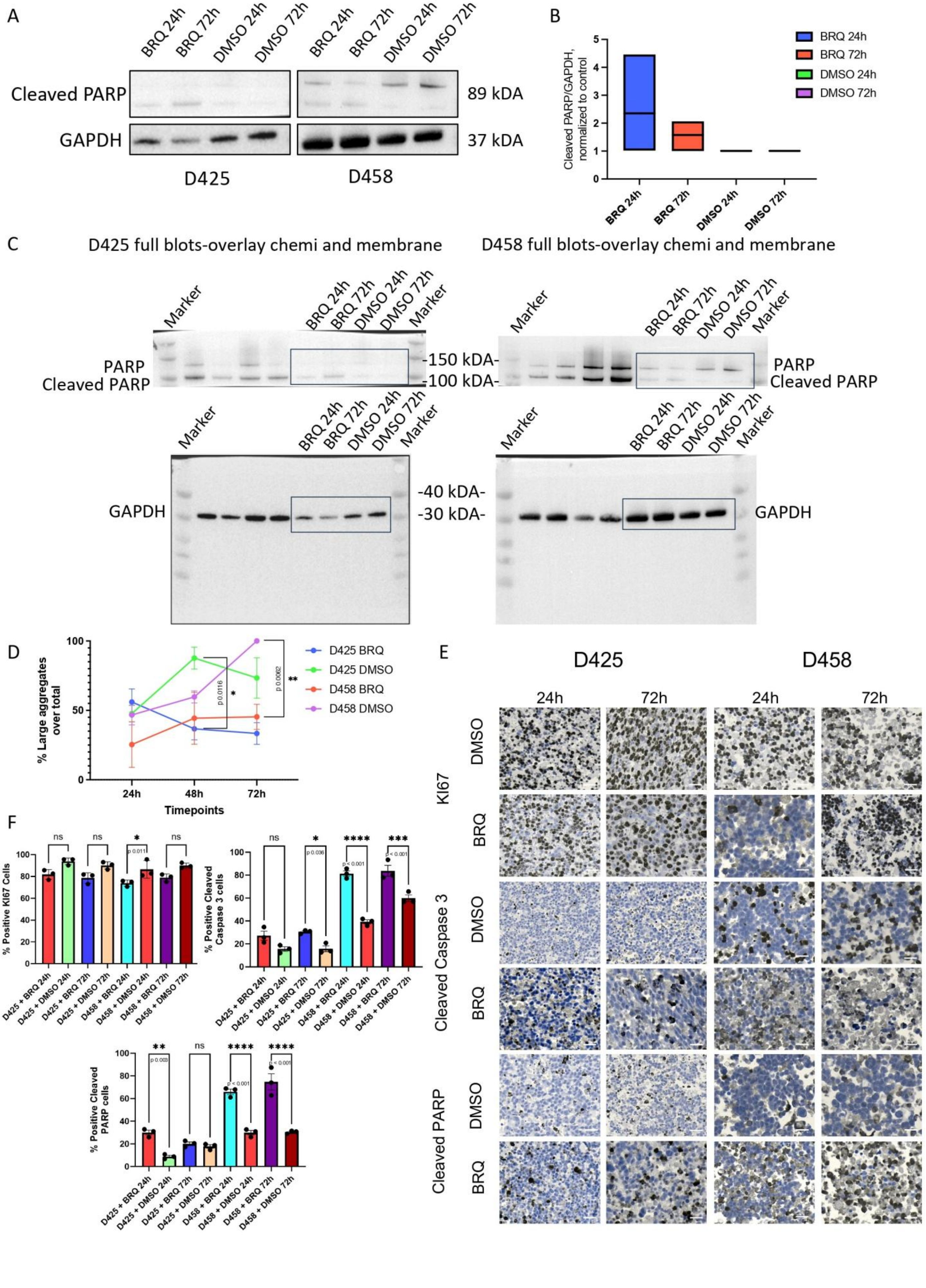
A
BRQ 24h
BRQ 72h
DMSO 24h
DMSO 72h
Cleaved PARP
GAPDH
89 kDA
37 kDA
D425
D458
B
Cleaved PARP/GAPDH, normalized to control
BRQ 24h
BRQ 72h
DMSO 24h
DMSO 72h
C
D425 full blots-overlay chemi and membrane
D458 full blots-overlay chemi and membrane
Marker
PARP
Cleaved PARP
-150 kDA-
-100 kDA-
-40 kDA-
-30 kDA-
GAPDH
D
% Large aggregates over total
Timepoints
24h
48h
72h
D425 BRQ
D425 DMSO
D458 BRQ
D458 DMSO
p 0.0116
p 0.0062
E
D425
D458
24h
72h
KI67
Cleaved Caspase 3
Cleaved PARP
DMSO
BRQ
F
% Positive KI67 Cells
% Positive Cleaved Caspase 3 cells
% Positive Cleaved PARP cells
ns
p 0.011
p 0.036
p < 0.001
p 0.003
D425 + BRQ 24h
D425 + DMSO 24h
D425 + BRQ 72h
D425 + DMSO 72h
D458 + BRQ 24h
D458 + DMSO 24h
D458 + BRQ 72h
D458 + DMSO 72h

Figure S2. Densiometric quantification of western blots. (**A**) Protein expression of selected markers in D425 and D458 cells after 24h and 72h treatment with single-treatment with BRQ IC50 or DMSO. The following proteins were assessed: cleaved-PARP (89 kDa) and the loading control GAPDH (37 kDa). The experiments were repeated at least three times with comparable results. Protein expression was determined by western blot analysis. (**B**) Densitometric analyses of protein bands from western blots using D425 and D458 cell lysates after 24h and 72h treatment with single-treatment with BRQ IC50 or DMSO, from at least three biological replicates. Proteins assessed cleaved-PARP (89 kDa) and the loading control GAPDH (37 kDa). Floating bars with median and min to max are shown. (**C**) Full blots displaying overlay chemiluminescence image and membrane image showing protein expression, from western blot analyses, in D425 and D458 cells after 24h and 72h treatment with single treatment with BRQ IC50 or DMSO. Proteins assessed were cleaved-PARP (89 kDa) and the loading control GAPDH (37 kDa). Marker was loaded and is shown on both sides of blots. (**D**) Quantification of D425 and D458 cellular aggregation at 24-, 48- and 72h after single-treatment with BRQ IC50 or DMSO, shown as the percentage of large aggregates over total number of aggregates. Aggregates are defined by cell number (3 or more cells clumped together, large aggregates consisting of >15 cells). (**E**) Immunohistochemistry staining for proliferating cells (Ki-67) and apoptosis/cell death (cleaved Caspase-3 and cleaved PARP) in D425 and D458 cell lines after 24 h or 72 h treatment with BRQ IC50 treatment or DMSO. Scale bars, 50 µm. (**F**) Immunohistochemical analyses showing % of cells positive for proliferation marker KI-67, cleaved Caspase-3 and cleaved PARP in D425 and D458 cell lines after 24- or 72h treatment with BRQ IC50 treatment or DMSO. Mean ± SD is presented. The number of % positive cells was determined using QuPath v0.4.3.

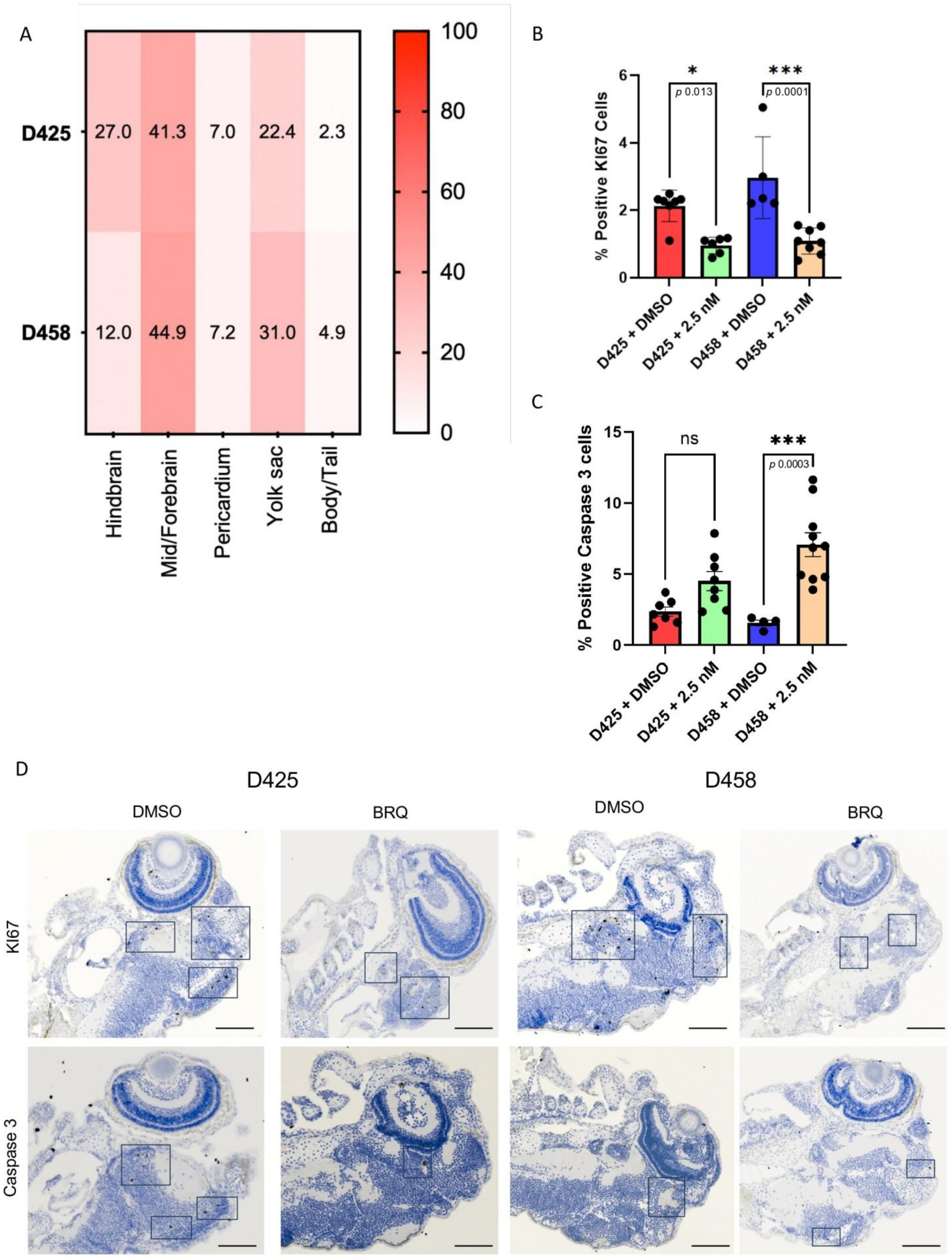
A
D425
D458
27.0
41.3
7.0
22.4
2.3
12.0
44.9
7.2
31.0
4.9
100
80
60
40
20
0
Hindbrain
Mid/Forebrain
Pericardium
Yolk sac
Body/Tail
B
% Positive KI67 Cells
*
p 0.013
***
p 0.0001
D425 + DMSO
D425 + 2.5 nM
D458 + DMSO
D458 + 2.5 nM
C
% Positive Caspase 3 cells
ns
***
p 0.0003
D425 + DMSO
D425 + 2.5 nM
D458 + DMSO
D458 + 2.5 nM
D
D425
D458
DMSO
BRQ
DMSO
BRQ
KI67
Caspase 3

Figure S3. Group 3 MB cell lines display preferential localization towards the brain area upon transplantation into 1K stage zebrafish embryos (**A**) Heatmaps of the tumour cell location of transplanted group 3 cell lines in 48 h old embryos. 1 = hindbrain area; 2 = mid/forebrain area; 3 = pericardium area; 4 = yolk sac area; 5 = body and tail area showing percentage localization. (**B**,**C**) Immunohistochemical analyses showing % of cells positive for proliferation marker KI-67 (B) and Caspase-3 (C) in transplanted zebrafish embryos after 72 h treatment with 2.5 nM BRQ treatment or DMSO. Mean ± SD is presented (D425 + Breq 2.5 nM n = 6, D425 + DMSO n = 5, D458 + Breq 2.5 nM n = 5, D458 + DMSO n = 8). The number of % positive cells was determined using QuPath v0.4.3. (**D**) Immunohistochemistry staining for proliferating cells (Ki-67) and Caspase-3 (used to validate the presence of transplanted human cells) in D425 and D458 cell lines after 24 h or 72 h treatment with BRQ IC50 treatment or DMSO. Scale bars, 50 μm.

Table S1. All cell lines, along with growth patterns, group classification, and other characteristics.

Table S2. Gene set enrichment analysis (GSEA).

Table S1–S2. are provided separately, attached as Excel files.
